# Effect of Monomer Feeding Strategy on the Sequence and Properties of Fluorine-Containing Polyarylates via Interfacial Polycondensation

**DOI:** 10.3390/polym18020267

**Published:** 2026-01-19

**Authors:** Lingli Li, Tiantian Li, Siyu Chen, Jintang Duan, Cailiang Zhang, Xueping Gu, Lianfang Feng

**Affiliations:** 1 Institute of Zhejiang University-Quzhou, Quzhou 324000, China; 2 State Key Laboratory of Chemical Engineering and Low-Carbon Technology, College of Chemical and Biological Engineering, Zhejiang University, Hangzhou 310027, China

**Keywords:** fluorine-containing polyarylates, interfacial polycondensation, monomer feeding strategy, process characteristics, properties

## Abstract

Fluorine-containing polyarylates (F-PARs) were synthesized via interfacial polycondensation of hexafluorobisphenol A (BPAF), bisphenol A (BPA), and two acyl chloride monomers under four feeding strategies. Sequential feeding affords the highest *M*_w_ (2.02 × 10^5^ g/mol) and high alternating sequence content; the one-pot method gives intermediate *M*_w_ and a random sequence; and segmented and parallel methods yield lower-*M*_w_ polymers and pseudo-block sequences. Time-resolved GPC results reveal that the concentration of -CF_3_-activated acyl chloride termini during chain propagation controls the subsequent chain propagation and, thus, the final *M*_w_. Consequently, sequential feeding delivers the highest *T*_g_ (215 °C) and stiffness (2.51 GPa) for thermal–mechanical loads; the one-pot protocol maximizes optical clarity (*T*_450_ = 85%) for transparent films. Systematic variation in the BPAF/BPA ratio via sequential feeding further reveals that higher BPAF content increases *M*_w_, enhances thermal stability, and blue-shifts UV absorption, whereas BPA-rich compositions improve the tensile strength and modulus. These findings provide a quantitative roadmap for the rational design of F-PAR chain architectures, enabling on-demand tuning of thermal, mechanical, and optical properties without additional synthetic complexity.

## 1. Introduction

Polyarylates (PARs) represent a notable class of high-performance polymers, valued for their superior heat resistance, mechanical performance, and low dielectric constant, which enable demanding applications in specialty films, structural fibers, and precision electronic devices [1,2,3,4]. Melt polycondensation is widely employed in industry for the production of liquid–crystalline PARs, owing to its high productivity and process efficiency, as well as the limited solubility of the resulting polymers in common solvents [5]. For bisphenol-based amorphous PARs, solution polycondensation and interfacial polycondensation (IP) are more commonly adopted. Among these approaches, IP offers advantages for achieving high molecular weights at ambient reaction conditions [6,7]. Typically, diacyl chlorides in an organic solvent only encounter bisphenolate ions in an aqueous phase at or near a dynamic interfacial region [8,9]. This spatial confinement of the reaction locations not only facilitates rapid polycondensation under mild conditions, but also suppresses side reactions inherent to melt polycondensation, rendering IP an important synthetic method for high-performance PARs [10,11].

Incorporation of fluorine-containing comonomers into PAR backbones, i.e., hexafluorobisphenol A (BPAF), can enhance their thermal stability and solubility and reduce the dielectric constant [12,13,14,15]. For instance, He et al. [16] demonstrated that introducing 5% BPAF increased the 5% weight-loss degradation temperature (*T*_d,5%_) by 71 °C compared to its bisphenol A (BPA)-based counterpart, highlighting the contribution of fluorine in reinforcing the polymer backbone against thermal degradation. In a more comprehensive study, Wang et al. [15] systematically replaced bisphenol B with BPAF and observed linear trends in property enhancement: the glass transition temperature (*T*_g_) rose from 201.6 to 237.1 °C, *T*_d,5%_ increased from 474.1 to 491.5 °C, and tensile strength varied from 52.63 to 59.93 MPa across the compositional series.

However, the macroscopic properties of fluorine-containing PAR (F-PAR) are not dictated by composition alone. The molecular weight and the sequence distribution of F-PARs exert significant influences as well. However, the copolymer sequence via IP is not a simple function of the bulk feed ratio but is instead governed by the local monomer stoichiometry and complex kinetics at the aqueous–organic interface [17]. Efforts to control sequences in copolycondensation have traditionally relied on monomer chemistry and the overall feed ratio of monomers. In IP, however, the feeding method, including reactant addition sequences and timing, can affect mass transfer and local interfacial concentrations, which potentially influence polycondensation kinetics. Wang et al. [18] prepared PARs with random and block molecular structures by one-pot feeding and sequential two-stage feeding, respectively. Nevertheless, a mechanistic understanding of how varied feeding methods influence the interfacial kinetic pathway to control the molecular weight, distribution, sequences, and properties of F-PARs remains a knowledge gap.

Here, four different monomer feeding methods, including one-pot, parallel, sequential, and segmented, were designed and evaluated for their efficacy in controlling the molecular weight and sequences of F-PARs via IP. The mechanism for the varied feeding method affecting the kinetic pathways was elucidated via time-resolved kinetic analysis and chain structure characterization. The thermal, optical, and mechanical properties of polymers prepared by different feeding methods were analyzed. Lastly, the effects of the BPAF/BPA ratio in sequential feeding on the resulting polymers’ molecular weight, sequences, and properties were investigated.

## 2. Materials and Methods

### 2.1. Materials

Terephthaloyl chloride (TPC, ≥99%), isophthaloyl chloride (IPC, ≥98%), BPAF (≥98%), BPA (≥99%), triethylbenzylammonium chloride (TEBAC, 98%), and deuterated dichloromethane (99.8% + 0.03% *v*/*v* tetramethylsilane) were supplied by Shanghai Macklin Biochemical Technology Co., Ltd. (Shanghai, China). Dichloromethane (DCM, ≥99.5%), anhydrous ethanol (≥99.7%), HCl (36.0–38.0%), and chloroform (≥99.0%) were obtained from Sinopharm Chemical Reagent Co., Ltd. (Shanghai, China). Sodium hydroxide (NaOH, ≥96%) was sourced from Xilong Scientific (Guangdong, China).

### 2.2. Synthesis of F-PARs via IP

**Figure 1:**
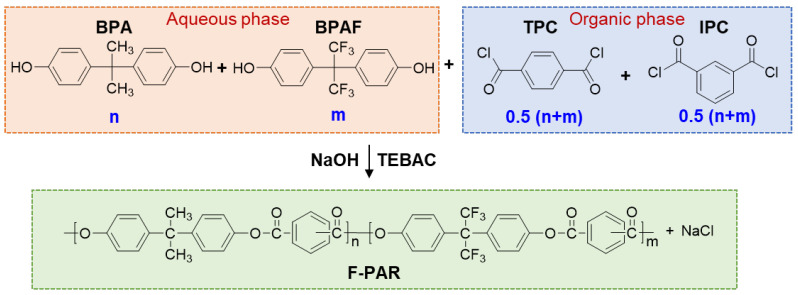
Schematic of F-PAR synthesis through interface polycondensation.

F-PARs were synthesized via IP in a glass reactor equipped with a mechanical stirrer. The general reaction scheme is illustrated in Figure 1. Unless otherwise specified, all reactions were conducted at 25 °C for a total duration of 4 h, using a water–DCM solvent system with TEBAC as a phase-transfer catalyst. A typical work-up procedure involved phase separation and purification following reaction completion. The organic phase was acidified with 10 mL of HCl (1 mol/L) to adjust the pH to 4–5. DCM was removed by azeotropic distillation with 50 mL of hot water at 80 °C. The resulting white solid was washed twice with hot water (80 °C) and anhydrous ethanol, successively, and dried at 120 °C for 1 h.

### 2.3. Specific Monomer Feeding Methodologies

**Figure 2:**
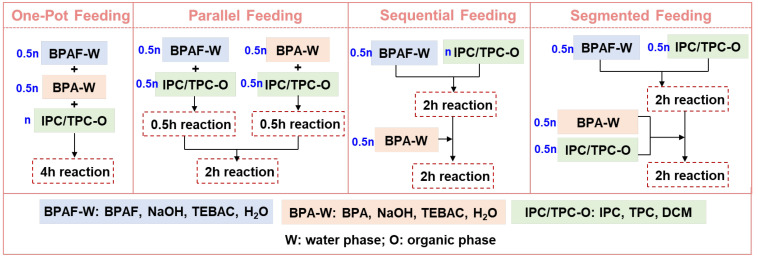
Summary of detailed procedures for four different feeding methods.

Four feeding methods (Figure 2) were designed to investigate their influence on the polycondensation process and the resulting polymer properties.

#### 2.3.1. One-Pot Feeding

This method served as the baseline control, involving the simultaneous addition of all monomers. BPA (13 mmol), BPAF (13 mmol), NaOH (54 mmol), and TEBAC (15 mg) were dissolved in 80 mL of deionized water. Due to the extremely low aqueous solubility of the diacyl chloride monomers (IPC and TPC), their contact with bulk water was restricted, kinetically hindering hydrolysis. Furthermore, hydrolysis of the diacyl chloride would typically lead to emulsion formation [19]. In our experiments, no such stable emulsion was observed. Therefore, while acyl chloride hydrolysis is a known side reaction, its influence in this work is considered negligible. Separately, IPC (12.5 mmol) and TPC (12.5 mmol) were dissolved in 60 mL of DCM. The organic solution was then mixed with the aqueous bisphenolate solution to initiate the polycondensation.

#### 2.3.2. Parallel Feeding

In this approach, the total monomers were partitioned to perform two parallel pre-reactions before final coupling. Two separate aqueous solutions were prepared: (I) BPAF (13 mmol), NaOH (27 mmol), and TEBAC (7.5 mg) in 40 mL of water; (II) BPA (13 mmol), NaOH (27 mmol), and TEBAC (7.5 mg) in 40 mL of water. The acyl chloride solution (IPC/TPC, 12.5 mmol each in 60 mL DCM) was also divided into two equal parts (30 mL each). Each bisphenolate solution was then combined with one portion of the acyl chloride solution to pre-react for 0.5 h. This specific pre-reaction duration was selected based on prior kinetic studies [20], which indicated that homopolycondensation of BPA and BPAF under analogous conditions reached weight-average molecular weights (*M*_w_) of approximately 3.48 × 10^4^ g/mol and 7.93 × 10^4^ g/mol, respectively, within 0.5 h. This stage yields oligomers with a high concentration of active chain ends, ideal for subsequent coupling. Extending this isolated pre-reaction to 2 h, where the *M*_w_ values plateau near 12.5 × 10^4^ g/mol (BPA) and 13.1 × 10^4^ g/mol (BPAF), would reduce end-group reactivity and compromise coupling efficiency. The two resulting oligomer mixtures were then combined in the main reactor and stirred for an additional 2 h.

#### 2.3.3. Sequential Feeding

This method involved the stepwise addition of two different bisphenolate solutions. For the standard 50/50 BPAF/BPA molar ratio system, aqueous solutions of BPAF and BPA were prepared as described in Section 2.3.2 (parallel feeding). Separately, IPC and TPC (12.5 mmol each) were dissolved in 60 mL of DCM. Solution I and the acyl chloride/DCM solution were reacted first for 2 h, followed by the addition of Solution II for the remaining 2 h.

To systematically investigate the influence of bisphenol composition, sequential feeding experiments were conducted across a range of BPAF/BPA molar ratios (from 90/10 to 10/90). In all cases, the total bisphenol amount was maintained at 26 mmol, and the total acyl chloride amount (IPC/TPC, 1:1 molar ratio) at 25 mmol. For each ratio, the acyl chloride solution was reacted first with the specified equivalent of BPAF. After 2 h of reaction, the BPA was introduced, and the polycondensation continued for an additional 2 h. All other reaction conditions remained consistent.

#### 2.3.4. Segmented Feeding

This method involved the divided addition of a complete set of monomers over two stages. Aqueous solutions of BPAF and BPA were prepared as described in Section 2.3.2 (parallel feeding). The acyl chloride solution (IPC/TPC, 12.5 mmol each in 60 mL DCM) was also divided into two equal parts. The reaction started with the addition of Solution I and the first half of the acyl chloride solution. After reacting for 2 h, Solution II and the remaining half of the acyl chloride solution were added simultaneously, and the reaction was allowed to proceed for a further 2 h.

### 2.4. Characterization

#### 2.4.1. Gel Permeation Chromatography (GPC)

The number-average molecular weights (*M*_n_) and *M*_w_ of the F-PARs were measured using an Agilent 1260 Infinity II GPC system (Agilent Technologies, Inc., Santa Clara, CA, USA). Chloroform served as the mobile phase. The measurements were performed by injecting sample solutions at a concentration of 3 mg/mL. The GPC system, calibrated with polystyrene standards, was capable of resolving polymers within a molecular weight range of 5 × 10^2^ to 10^6^ g/mol. The system was calibrated with polystyrene (PS) standards. PDI was calculated as the ratio of *M*_w_ to *M*_n_. It should be noted that the rigid backbone and fluorine substituents of F-PARs likely result in a different hydrodynamic volume-to-molecular weight relationship compared to flexible PS chains. Therefore, the reported *M*_w_ values should be treated as apparent molecular weights relative to PS standards. Nevertheless, since all samples share the same chemical structure and were analyzed under the same GPC conditions, the relative trends remain valid for comparing polycondensation efficiency and chain-growth behavior.

#### 2.4.2. ^1^H Nuclear Magnetic Resonance (NMR) Spectroscopy

^1^H NMR spectra were acquired on a Bruker AVANCE III 500 MHz spectrometer (Bruker Corporation, Billerica, MA, USA). The measurements were performed at 25 °C using deuterated dichloromethane as the solvent. Sample concentrations were 50 mg per 0.6 mL of solvent.

#### 2.4.3. Differential Scanning Calorimetry (DSC)

DSC measurements were conducted on a TA instrument, DSC 250 (TA Instruments, New Castle, DE, USA), under nitrogen. Samples were subjected to the following thermal program: heating from 50 to 300 °C at 10 °C/min, isothermal holding for 10 min, cooling to 50 °C at 10 °C/min, another isothermal hold for 10 min, and a second heating scan to 300 °C at 10 °C/min. *T*_g_ was determined from the second heating scan.

#### 2.4.4. Thermo Gravimetric Analysis (TGA)

The thermal stability of the polymers was assessed by TGA on a Mettler-Toledo TGA/DSC 3+ instrument (Mettler-Toledo International Inc., Greifensee, Switzerland). The samples were heated from 50 to 700 °C at 20 °C/min under nitrogen.

#### 2.4.5. Transmittance Analysis

The optical transparency of the solution-cast films was characterized by ultraviolet–visible (UV–Vis) spectroscopy. Transmittance spectra were recorded in the 200–700 nm wavelength range using an Agilent Cary 5000 spectrophotometer (Agilent Technologies, Inc., Santa Clara, CA, USA). The transmittance (*T*) was calculated from the absorbance (*A*) using the formula *T* = 10^−^*^A^*.

#### 2.4.6. Mechanical Tensile Testing

The tensile properties were characterized using a SUNS universal testing machine (SUNS Industrial Group Co., Ltd., Shenzhen, China). Specimens (3-type dumbbell-shaped, GB/T 528-2009 [21]) were cut from solution-cast films and tested at 25 °C with a speed of 5 mm/min. A minimum of five replicates per sample were tested.

## 3. Results and Discussion

### 3.1. Effect of Feeding Method

#### 3.1.1. Molecular Chain Structure Characterization

**Figure 3:**
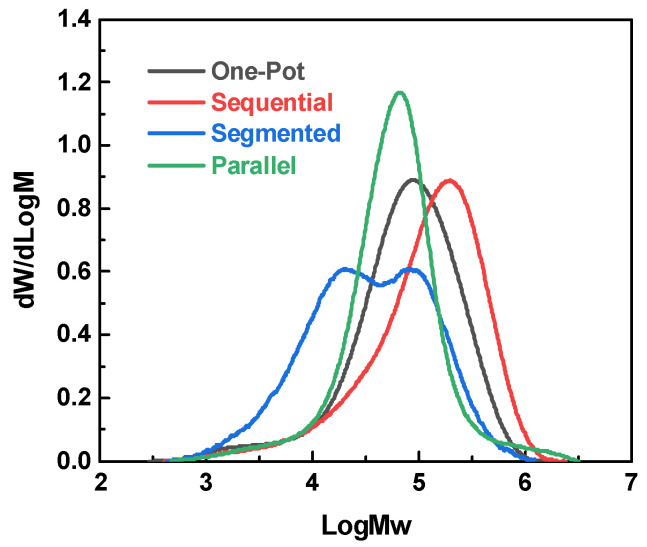
GPC curves of the F-PARs prepared by various feeding methods.

Table 1 summarizes the *M*_n_, *M*_w_, and PDI of F-PARs prepared under four feeding methods, and the GPC elution curves are shown in Figure 3. The one-pot method yields a polymer with *M*_w_ = 1.26 × 10^5^ g/mol and PDI = 5.09, values that serve as the benchmark for the subsequent comparisons. Parallel feeding reduces *M*_w_ to 1.05 × 10^5^ g/mol but with a lower PDI. The segmented method gives the lowest *M*_w_ (7.4 × 10^4^ g/mol), while the sequential method yields the highest *M*_w_ (2.03 × 10^5^ g/mol). Furthermore, the molecular weight distribution for the polymers synthesized via the one-pot, sequential, and parallel methods is unimodal, whereas the product obtained through the segmented feeding process exhibits a bimodal distribution.

In the one-pot method, simultaneous addition of all monomers is expected to generate a random copolycondensation of BPA and BPAF. In the parallel method, BPAF and BPA first react separately with stoichiometric acyl chlorides. Our previous study [20] showed that these reactions reached *M*_w_ values of 7.9 × 10^4^ (BPA) and 3.5 × 10^4^ g/mol (BPAF) within 30 min. During the subsequent 2 h reaction, the two types of polymer chains continue propagating, resulting in a unimodal and narrower distribution. The segmented method follows a two-stage process where BPAF is nearly exhausted in the first 2 h, followed by the addition of BPA and fresh acyl chloride, which primarily initiates BPA homopolycondensation accompanied by chain propagation of early-stage polymers. This process generates two polymer populations, accounting for the bimodal distribution and the lowest *M*_w_. Conversely, the sequential method uses an excess of acyl chloride during the initial BPAF reaction, favoring the formation of IPC-BPAF-TPC oligomers rather than long chains. Once BPA is introduced, these oligomers are rapidly connected to increase chain length. Incorporation of BPAF in the oligomers increases the electrophilicity of the acyl chloride terminals per our previous electrostatic potential (ESP) analysis [20], resulting in the highest *M*_w_, which will be further discussed with time-resolved analysis.

**Figure 4:**
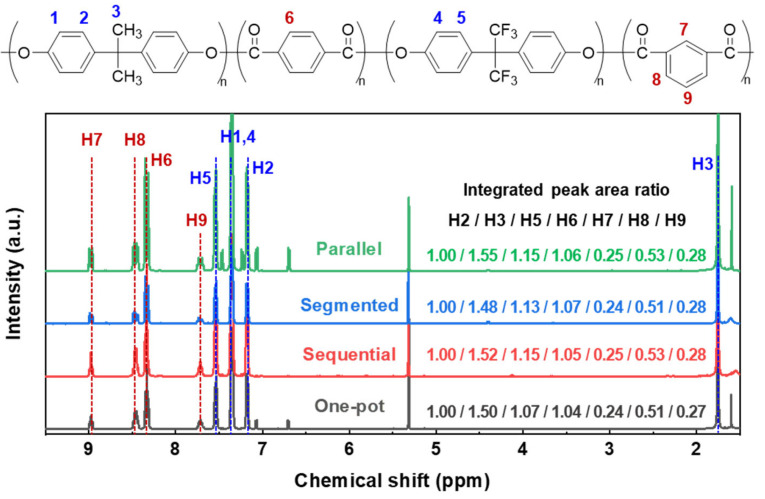
^1^H NMR spectra of the F-PARs prepared by various feeding methods.

The sequences of the copolymers were determined by ^1^H NMR spectroscopy (Figure 4). The integrated peak area ratio of H2:H3:H5:H6:H7:H8:H9 is approximately 4:6:4:4:1:2:1, which aligns well with the expected monomer feed ratio of BPA:BPAF:IPC:TPC = 1.04:1.04:1.00:1.00. This confirms the consistency between the feed stoichiometry and the copolymer composition. The H2 and H3 signals originate from BPA, while the H5 signal arises from BPAF. The integrated peak area ratio of H2:H5 is close to 1:1.1 for all feeding methods, indicating a slightly higher incorporation of BPAF than BPA in the resulting copolymers. The presence of the -CF_3_ group in the copolymers was further confirmed by ^19^F NMR spectroscopy (Figure S1), which exhibits a characteristic single resonance at *δ* −64.1 ppm. Complementary structural insight was obtained from ^13^C NMR spectroscopy (Figure S2). The spectrum displays characteristic signals for the ester carbonyl carbons at *δ* 162.8 and 164.3 ppm; the -CF_3_ carbon quartet at *δ* 120.8, 123.0, 125.3, and 127.6 ppm; the methyl carbons of BPA at *δ* 30.6 ppm; and the quaternary carbon adjacent to the -CH_3_ groups at *δ* 42.5 ppm. However, due to partial baseline overlap and signal broadening, a reliable quantitative analysis of these peaks in the ^13^NMR spectrum was not feasible. Together, the ^1^H, ^19^F, and ^13^C NMR spectra provide robust spectroscopic evidence for the copolymer structure.

**Figure 5:**
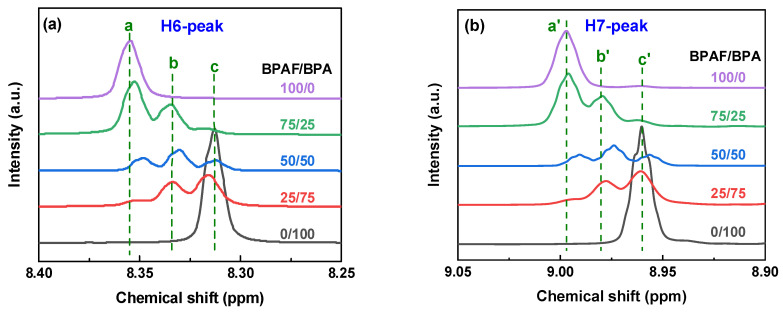
^1^H NMR spectra for H6-peak (**a**) and H7-peak (**b**) of the F-PARs with different BPAF/BPA ratios prepared by one-pot methods.

To decipher the sequence-level microstructure, the fine features of the ^1^H NMR spectra were examined. The H6 signal originates from a symmetric TPC aromatic ring, and H7, H8, and H9 correspond to the IPC aromatic ring. Triplet splitting in both H6 and H7 is observed, indicating whether the acyl chloride is connected to BPA or BPAF. The triads are designated as BPAF-A-BPAF, BPA-A-BPA, and BPAF-A-BPA (A = TPC or IPC). The respective assignments were determined by analysis of one-pot feeding samples with varying BPA and BPAF ratios (Figure 5). Spectral evolution tracks copolymer composition through a systematic, shift-correlated trend. The downfield triplets at *δ* 8.354 and 8.992 (a/a′), diagnostic of BPAF-A-BPAF triads, decrease monotonically with a decreasing BPAF ratio. The upfield triplets at *δ* 8.313 and 8.960 (c/c′), characteristic of BPA-A-BPA triads, intensify. Signals at *δ* 8.334 and 8.975 (b/b′) emerge only in mixed-bisphenol systems and are, therefore, assigned to BPAF-A-BPA hetero-triads. The quantitative relationship between these three signal sets validates their sequential origin and allows for direct spectroscopic quantification of triad contents. It should be noted that the quantification of triad contents via peak deconvolution and integration carries inherent uncertainties, primarily due to potential baseline artifacts and minor peak overlap in complex copolymer spectra. Nevertheless, the clear resolution of the diagnostic triplet signals and, more importantly, their consistent and monotonic evolution across a controlled compositional series (Figure 5) provide high confidence in the relative trends and comparisons.

The sequence distribution of each sample of different feeding methods was quantified by integrating the resolved sub-peaks of H6/H7 relative to their total intensity; the results are shown in Table 2. Generally, the sequential method generates the highest BPAF-A-BPA triad content (62%), followed by the one-pot (47.6%), parallel (9.4%), and segmented (8.8%) methods. In the one-pot experiment, both BPA and BPAF monomers simultaneously diffuse into the organic phase to react with acyl chlorides. Although their diffusion coefficients and reactivities differ, the instantaneous random copolycondensation produces a statistically random sequence. Higher BPAF-A-BPA triad content in the sequential method confirms a predominantly alternating microstructure. The significantly lower alternating triad contents in the parallel and segmented methods signify a pseudo-block architecture. The parallel method, where BPA and BPAF are allowed to react separately with acid chlorides before the streams are merged, generates longer fluorine-free segments than the segmented method, in which the bisphenols are introduced in discrete stages.

#### 3.1.2. Structure–Property Relationships Governed by Feeding Methods

**Figure 6:**
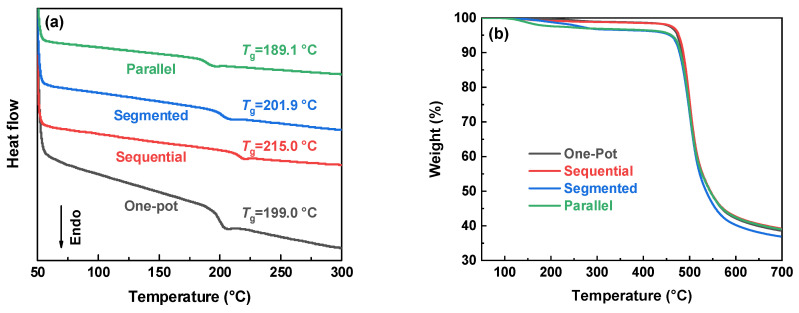
Thermal properties of the F-PARs prepared by various feeding methods: (**a**) DSC and (**b**) TGA curves.

Thermal, optical, and mechanical data for the four feeding methods are collated in Table 3. Figure 6 shows that the sequential sample exhibits the highest *T*_g_ (214.9 °C) and the highest initial decomposition temperatures (*T*_d,5%_ = 476.1 °C), stemming from its highest *M*_w_ and highest fraction of alternating sequences. In contrast, the parallel method displays the lowest *T*_g_ (189.1 °C) and a markedly reduced *T*_d,5%_ (459.7 °C). Segmented and one-pot products give intermediate and virtually identical *T*_g_ values (201.9 and 199.0 °C, respectively). However, the segmented polymer shares the poor thermal stability of the parallel analogue, whereas the one-pot sample is substantially more stable. Collectively, these results indicate the length and the fraction of fluorine-free segments as the primary determinants of thermal properties.

Optical clarity follows the order segmented < parallel < sequential < one-pot, aligning with the extent of sequence disorder (Figure S3). In aromatic copolymers, the degree of sequence regularity plays an important role in molecular stacking and π–π interactions [22]. The random copolymer (one-pot) distributes -CF_3_ and -CH_3_ segments statistically, disrupting π-π stacking and minimizing charge–transfer interactions; consequently, it delivers the highest visible-light transmittance, yet its *λ*_cut-off_ remains at 321 nm because residual short BPA-rich blocks still permit some inter-chain ordering. The pseudo-alternating architecture generated by the sequential method increases main-chain twisting and inter-molecular free volume, pushing *λ*_cut-off_ to the lowest value (315 nm) and yielding optical clarity slightly lower than that of the random material. Pseudo-block samples (parallel and segmented) assemble extended fluorine-free domains that can pack efficiently; the resulting stronger π-π interactions shift *λ*_cut-off_ to 321–322 nm and simultaneously reduce visible transmittance. Thus, alternation minimizes the absorption edge, whereas randomness maximizes transmission in the visible window; both outperform architectures that contain long BPA-A-BPA sequences.

Mechanical properties vary marginally with feeding method (Figure S4). Tensile strength falls within a narrow range of 60.9–63.0 MPa, indicating that differences in the molecular weight and the sequence may not perturb the entanglement network under the conditions tested. In contrast, the alternating-rich, sequential film is the stiffest (2.51 GPa), 11% higher than the quasi-block parallel sample (2.24 GPa). This modest stiffening is consistent with denser chain packing and reduced segmental mobility imposed by the regular BPAF-A-BPA motif.

Thus, tailoring the monomer feeding method allows the same two bisphenols to be directed toward distinct performance windows: the sequential route delivers the highest *T*_g_ and stiffness for thermal–mechanical loads; the one-pot protocol maximizes optical clarity for transparent films; and the parallel or segmented methods offer lower melt viscosity and broader processing windows, trading off some thermal stability and optical transparency for easier fabrication and intermediate strength.

### 3.2. Kinetic Superiority of Sequential Feeding: A Time-Resolved Study

**Figure 7:**
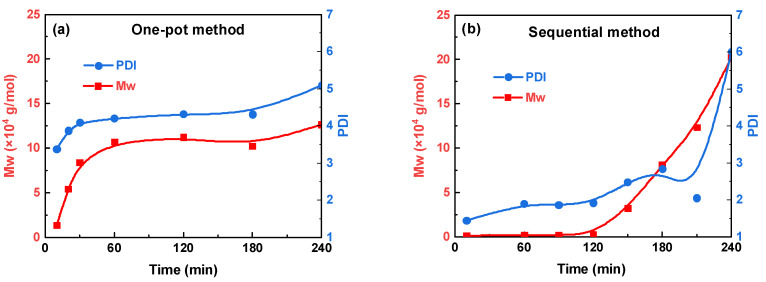
*M*_w_ and PDI evolution over time for the one-pot (**a**) and sequential (**b**) methods.

**Figure 8:**
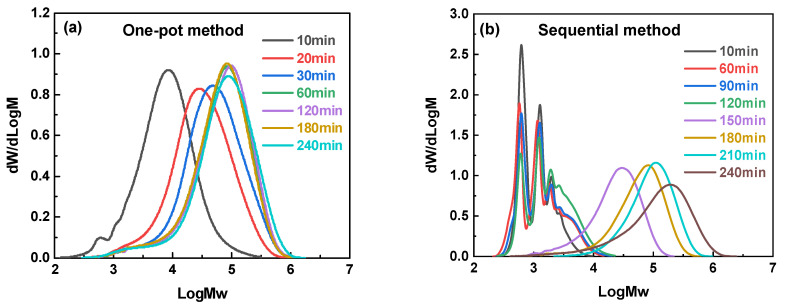
Time-dependent evolution of GPC profiles for the one-pot (**a**) and sequential (**b**) methods.

The molecular weight evolution over time for both the sequential and one-pot methods was monitored to probe the reasons for the differences in chain growth. One-pot polycondensation follows a rapid, self-limiting trend (Figure 7a). Within 10 min, the chains have already reached 1.3 × 10^4^ g/mol; after 60 min, the growth stalls at 8–11 × 10^4^ g/mol, and the dispersity remains unimodal (Figure 8a). The simultaneous presence of both bisphenols drives early fast propagation, but the finite inventory of acyl chloride and the statistical dilution of chain ends quickly exhaust the reaction, leading to an upper plateau.

In contrast, sequential feeding involves a two-stage evolution (Figure 7b). During the first 120 min, only BPAF is added, and the acyl chlorides are kept in one-fold excess. Short, acyl chloride-capped oligomers accumulate (<2500 g/mol, Figure 8b). The terminal -COCl groups are further activated by the adjacent -CF_3_ substituents, which exert a strong electron-withdrawing inductive effect, thereby increasing carbonyl electrophilicity [20,23,24,25]. Electrostatic potential analysis [20] supports this mechanism, revealing an elevated local electrostatic potential along BPAF-rich oligomers that facilitates nucleophilic attack. Beyond this electronic effect, the increased backbone rigidity induced by the bulky -CF_3_ groups may promote a more extended conformation of the oligomers at the interface [26], enhancing chain-end availability. Furthermore, the hydrophobicity and interfacial activity of the fluorinated oligomers may change their partitioning and local accumulation at the interface [27], thereby contributing to the observed polycondensation kinetics. When BPA is introduced at 120 min, the reaction system contains highly activated “seed” chains. A rapid second-stage propagation occurs, and the GPC curves coalesce into a single, narrow peak. The final *M*_w_ (2.03 × 10^5^ g/mol) almost doubles the one-pot plateau.

**Figure 9:**
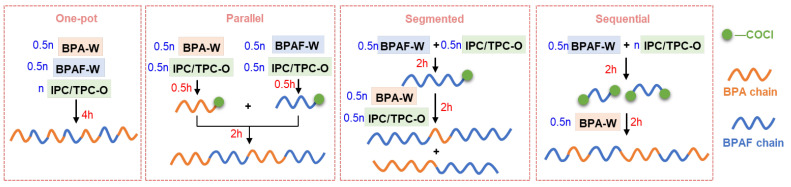
Schematic illustration of the chain-growth mechanisms for different monomer feeding methods.

The decisive factor is, therefore, the concentration of -CF_3_-activated acyl chloride termini during chain propagation among different feeding methods, as schematically illustrated in Figure 9. One-pot feeding produces them randomly and, therefore, reaches an upper plateau relatively early, leading to a relatively homogeneous, unimodal molecular weight distribution. Both parallel and segmented methods keep BPAF and acyl chlorides roughly stoichiometric in the first stage, generating longer chains but fewer activated acyl chloride termini and, therefore, a lower *M*_w_. The segmented method, however, involves a prolonged first-stage reaction that nearly exhausts the BPAF monomer. Subsequent introduction of BPA and fresh acyl chloride primarily initiates BPA homopolycondensation alongside limited chain extension of existing polymers, ultimately producing two distinct polymer populations and accounting for the observed bimodal molecular weight distribution. In contrast, the sequential feeding method first generates highly reactive “seed” oligomers, which then undergo rapid chain extension in the second stage, leading to the highest *M*_w_.

### 3.3. Effect of BPAF/BPA Ratios via Sequential Method

Using the sequential method, a range of copolymers with BPAF/BPA molar ratios varying from 90/10 to 10/90 were synthesized to investigate the effect of monomer ratios on the molecular chain structures and properties. Since the sequential feeding method requires two different bisphenol species to be added in separate stages, data for pure BPA or BPAF homopolymers are not included in this comparison.

#### 3.3.1. Molecular Chain Structure Characterization

**Figure 10:**
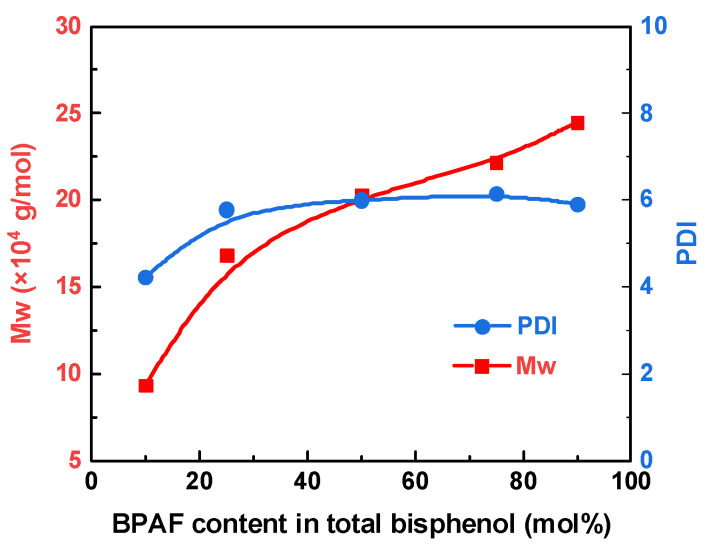
*M*_w_ and PDI of F-PARs with different BPAF/BPA molar ratios prepared by sequential methods.

Figure 10 shows the effect of composition on the molecular weight of F-PARs. The *M*_w_ increases from 9.3 × 10^4^ to 2.45 × 10^5^ g/mol as the BPAF content rises from 10% to 90%. For the 10/90 BPAF/BPA system, only 10% of the total bisphenol (BPAF) is reacted with 100% of the acyl chlorides in the first stage. This results in a nine-fold excess of acyl chlorides relative to the available bisphenol during this stage. Consequently, only a limited population of short, acid-chloride-capped oligomers is formed, leaving the majority of acyl chloride monomers unreacted and inactivated. Therefore, the *M*_w_ is relatively low when BPA is added at the second stage. As the BPAF content increases up to 90%, all -COCl termini are activated by -CF_3_ substituents, and the chain length of oligomers increases. When BPA is added later, it serves as a connector of the long-chain oligomers, and thus, a high *M*_w_ is achieved. Therefore, increasing BPAF progressively changes the role of acid chloride from “scarce initiator” to “oligomer cap” and finally to “long-chain cap”, switching the growth mode from site-starved termination to chain extension and yielding the observed molecular-weight increase.

**Figure 11:**
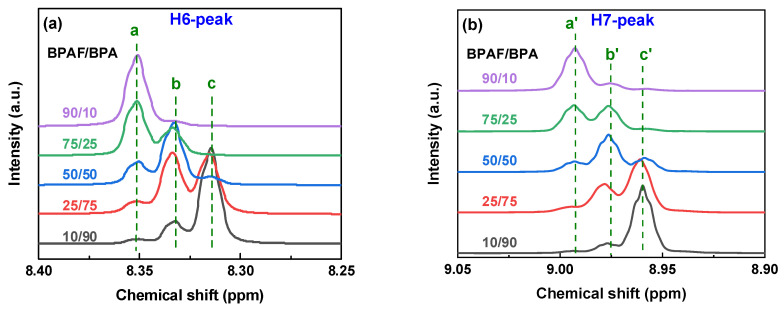
^1^H NMR spectra for H6-peak (**a**) and H7-peak (**b**) of F-PARs with different BPAF/BPA molar ratios prepared by sequential methods. a/a′, b/b′, and c/c′ peaks are characteristics of BPAF-A-BPAF, BPAF-A-BPA, and BPA-A-BPA triads, respectively.

Figure 11 and Table 4 summarize how the BPAF/BPA feed ratio tunes triad distribution. At 10/90 BPAF/BPA, the BPA-A-BPA triad dominates (80.9%). Raising the BPAF fraction to 50/50 maximizes the alternating BPAF-A-BPA triad (62%), whereas a 90/10 feed inverts to an 88.0% BPAF-A-BPAF triad. Interestingly, BPAF-TPC-BPAF contents are higher than BPAF-IPC-BPAF contents across all feeding ratios, and BPA-TPC-BPA contents are always lower than BPA-IPC-BPA contents, indicating a higher affinity between TPC and BPAF than between IPC and BPA.

#### 3.3.2. Properties as a Function of Chain Architecture

**Figure 12:**
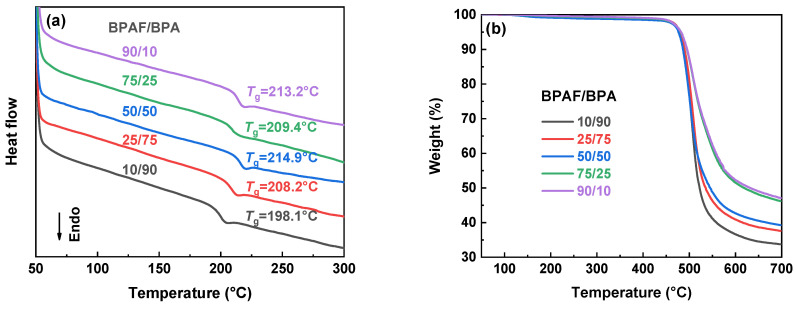
Thermal properties of F-PARs with different BPAF/BPA molar ratios prepared by sequential methods: (**a**) DSC and (**b**) TGA curves.

The properties of the F-PARs, summarized in Table 5, reveal correlations with the BPAF/BPA composition across thermal, optical, and mechanical metrics. As the BPAF/BPA molar ratio rises from 10/90 to 90/10, the *T*_g_ increases from 198.1 °C to 213.2 °C, and the initial decomposition temperatures (*T*_d,5%_ and *T*_d,10%_) show a corresponding rise (Figure 12). This improved thermal stability can be rationalized by the higher bond energy of the C–F bond (≈485 kJ·mol^−1^) relative to typical C–C, C–O, and C–H bonds present in the polymer backbone [15], which effectively restrains segmental mobility and delays the initial stage of degradation. Moreover, the residual char yield at 700 °C increases from 33.7% to 47.1%. The enhanced char-forming ability originates from the -CF_3_ groups. The strong electronegativity of fluorine lowers the bond-dissociation energy of the adjacent C–C bond in the –C–CF_3_ moiety, facilitating its cleavage to yield •CF_3_ radicals. These radicals efficiently capture other reactive radical species generated during polymer degradation, thereby promoting cross-linking reactions and enhancing char-forming [28]. These results demonstrate that incorporating more BPAF units enhances the overall thermal stability and char-forming ability of the copolymers.

Optical behavior correlates inversely with BPAF content (Figure S5). Films rich in BPAF (≥50%) maintain high transmittance (*T*_450_ = 81–83%), while BPA-rich films reduce it (*T*_450_ = 73–75%). Furthermore, the UV cut-off wavelength blue-shifts from 327 nm to 311 nm with an increasing BPAF fraction, indicating a widened optical band gap. Tensile performance shows a contrasting trend, strengthening with higher BPA content (Figure S6). Tensile strength increases by over 45% (43.8 to 63.7 MPa) and elastic modulus by nearly 50% (1.70 to 2.50 GPa) as BPA becomes the major component, suggesting that BPA units foster stronger intermolecular cohesion than their fluorine-containing counterparts.

## 4. Conclusions

In summary, the monomer feeding strategy serves as a powerful tool to dictate the interfacial polycondensation pathway, molecular chain architecture, and properties of F-PARs. Among the four methods evaluated, the sequential method stands out with a unique two-stage mechanism. The initial formation of CF_3_-activated oligomers, followed by a rapid second-stage propagation upon BPA addition, yields the highest *M*_w_ and a predominantly alternating sequence. This specific architecture contributes to superior thermal stability and material stiffness. In contrast, one-pot polycondensation follows a rapid, self-limiting trend with a relatively early upper plateau, producing a statistically random copolymer with optimal optical transparency. The parallel and segmented methods keep BPAF and acyl chlorides roughly stoichiometric in the first stage, generating longer chains but fewer activated acyl chloride termini. This leads to pseudo-block architectures with a lower *M*_w_, resulting in compromised thermal stability and optical clarity, albeit with potential processing advantages.

Using the sequential method, the BPAF/BPA composition affects the chain architecture and, thus, the final material properties. Increasing BPAF content from 10% to 90% increases the *M*_w_ from 9.3 × 10^4^ to 2.45 × 10^5^ g/mol and shifts the dominant triad sequence from BPA-A-BPA (80.9%) to alternating BPAF-A-BPA (62%) and finally to BPAF-A-BPAF (88.0%). Higher BPAF content increases *M*_w_, enhances thermal stability, and blue-shifts UV absorption, whereas BPA-rich compositions improve tensile strength and modulus. The resulting structure–property relationship matrix offers a predictive toolkit for tailoring F-PAR architecture, modulating thermal, mechanical, and optical properties without extra synthetic steps.

## Figures and Tables

**Table 1:** GPC data of the F-PARs prepared by various feeding methods.

Properties	Feeding Method			
	One-Pot	Parallel	Sequential	Segmented
*M*_w_ (10^4^ g/mol)	12.7	10.5	20.3	7.4
*M*_n_ (10^4^ g/mol)	2.5	2.7	3.4	1.4
PDI	5.09	3.88	6.00	5.40

**Table 2:** Sequence distribution of the F-PARs prepared by various feeding methods using a BPAF/BPA molar ratio of 50/50.

Triad Type	Triad Content (%)			
	One-Pot	Parallel	Sequential	Segmented
BPAF-IPC-BPAF	12.0	21.7	8.4	24.3
BPAF-IPC-BPA	23.4	5.9	29.5	3.2
BPA-IPC-BPA	12.5	20.6	11.1	19.9
BPAF-TPC-BPAF	15.3	26.7	13.7	25.3
BPAF-TPC-BPA	24.1	3.5	32.5	5.6
BPA-TPC-BPA	12.7	21.7	4.8	21.8

**Table 3:** Properties of the F-PARs prepared by various feeding methods.

Properties	Feeding Method			
	One-Pot	Parallel	Sequential	Segmented
*T*_g_ (°C)	199.0	189.1	214.9	201.9
*T*_d,5%_(°C) ^1^	473.2	459.7	476.1	456.2
*T*_d,10%_(°C) ^2^	483.9	480.0	486.3	477.9
*T*_d,max_ (°C) ^3^	502.7	500.2	501.5	501.4
Residual char yield at 700 °C (%)	38.6	39.0	39.3	36.9
*T*_400_ (%) ^4^	82.6	77.1	79.8	71.0
*T*_450_ (%) ^5^	85.5	80.6	82.6	78.1
λ_cut-off_ (nm) ^6^	321	322	315	321
Tensile strength (MPa)	60.9 ± 1.5	63.0 ± 0.5	61.2 ± 1.3	62.7 ± 1.7
Modulus of elasticity (GPa)	2.30 ± 0.07	2.24 ± 0.09	2.51 ± 0.08	2.37 ± 0.05

^1^ *T*_d,5%_: thermal decomposition temperatures at 5% weight loss; ^2^ *T*_d,10%_: thermal decomposition temperatures at 10% weight loss; ^3^ *T*_d,max_: thermal decomposition temperatures at maximum weight loss rate; ^4^ *T*_400_: transmittance at the wavelength of 400 nm; ^5^ *T*_450_: transmittance at the wavelength of 450 nm; ^6^ *λ*_cut-off_: UV cut-off wavelengths.

**Table 4:** Sequence distribution of F-PARs with different BPAF/BPA molar ratios prepared by sequential methods.

	BPAF/BPA	Triad Content (%)				
Triad Type		90/10	75/25	50/50	25/75	10/90
BPAF-IPC-BPAF		41.5	23.3	8.5	3.4	1.6
BPAF-IPC-BPA		6.2	22.1	29.9	15.1	5.8
BPA-IPC-BPA		2.1	2.8	10.7	30.1	41.0
BPAF-TPC-BPAF		46.5	33.9	13.9	5.5	2.5
BPAF-TPC-BPA		3.3	16.6	32.3	22.5	9.2
BPA-TPC-BPA		0.5	1.4	4.6	23.3	39.9

**Table 5:** Properties of the F-PARs with different BPAF/BPA molar ratios prepared by sequential methods.

Properties	BPAF/BPA Molar Ratio				
	90/10	75/25	50/50	25/75	10/90
*T*_g_ (°C)	213.2	209.4	214.9	208.2	198.1
*T*_d,5%_ (°C)	481.8	481.0	476.1	478.7	477.7
*T*_d,10%_ (°C)	495.4	494.9	486.3	489.6	487.4
*T*_d,max_ (°C)	506.6	507.7	501.5	507.5	500.3
Residual char yield (%)	47.1	46.2	39.3	37.6	33.7
*T*_400_ (%)	79.7	76.4	79.8	67.0	65.1
*T*_450_ (%)	82.6	81.1	82.6	74.8	73.7
*λ*_cut-off_ (nm)	311	317	315	326	327
Tensile strength (MPa)	43.8 ± 1.9	52.1 ± 1.4	61.2 ± 1.3	62.3 ± 2.2	63.7 ± 1.4
Modulus of elasticity (GPa)	1.70 ± 0.12	2.08 ± 0.08	2.51 ± 0.08	2.45 ± 0.09	2.50 ± 0.01

## Data Availability

The original contributions presented in this study are included in the article. Further inquiries can be directed to the corresponding author.

## Supplementary Materials

The following supporting information can be downloaded at https://www.mdpi.com/article/10.3390/polym18020267/s1: Figure S1: ^19^F NMR spectra of the F-PARs with 50/50 BPAF/BPA molar ratios prepared by sequential feeding methods; Figure S2: ^13^C NMR spectra of the F-PARs with 50/50 BPAF/BPA molar ratios prepared by sequential feeding methods; Figure S3: UV-Vis transmittance spectra of the F-PARs prepared by various feeding methods; Figure S4: Stress-strain curves of the F-PARs prepared by various feeding methods; Figure S5: UV-Vis transmittance spectra of F-PARs with different BPAF/BPA molar ratios prepared by sequential methods; Figure S6: Stress-strain curves of F-PARs with different BPAF/BPA molar ratios prepared by sequential methods.
