# Supplementary material for: Effect of Monomer Feeding Strategy on the Sequence and Properties of Fluorine-Containing Polyarylates via Interfacial Polycondensation
Source: Polymers (Basel). 2026 Jan 19;18(2):267. doi: 10.3390/polym18020267 (PMC12845985; doi:10.3390/polym18020267)
Supplement: Supplementary file 1 [file polymers-18-00267-s001.zip › polymers-4093006-supplementary.pdf]

polymers

Effect of Monomer Feeding Strategy on the Sequence and Properties of Fluorine-Containing Polyarylates via Interfacial Polycondensation

Lingli Li [1,†], Tiantian Li [1,2,†], Siyu Chen [1,2], Jintang Duan [1,2,*], Cailiang Zhang [1,2], Xueping Gu [1,2] and Lianfang Feng [1,2]

[1] Institute of Zhejiang University-Quzhou, Quzhou 324000, China; lilingli@zju.edu.cn (L.L.); li-tiantian@zju.edu.cn (T.L.); chen_yolo@zju.edu.cn (S.C.); zhangcailiang@zju.edu.cn (C.Z.); guxueping@zju.edu.cn (X.G.); fenglf@zju.edu.cn (L.F.)

[2] State Key Laboratory of Chemical Engineering and Low-Carbon Technology, College of Chemical and Biological Engineering, Zhejiang University, Hangzhou 310027, China

* Correspondence: duanj@zju.edu.cn; Tel.: +86-570-8015-102; Fax: +86-570-8015102

† These authors contributed equally to this work.

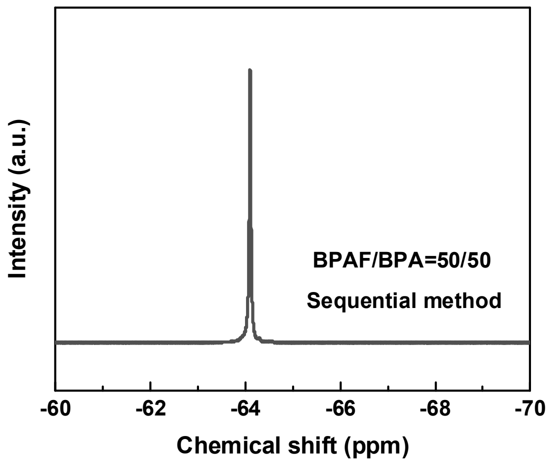

Figure S1. ^{19}F NMR spectra of the F-PARs with 50/50 BPAF/BPA molar ratios prepared by sequential feeding methods.

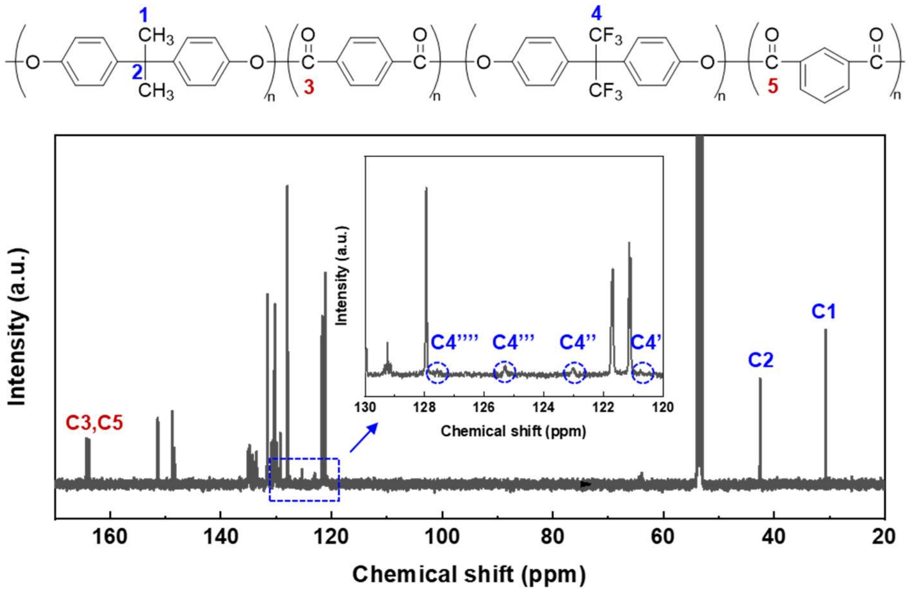

 https://doi.org/10.3390/polym18020267

Figure S2. ^{13}C NMR spectra of the F-PARs with 50/50 BPAF/BPA molar ratios prepared by sequential feeding methods.

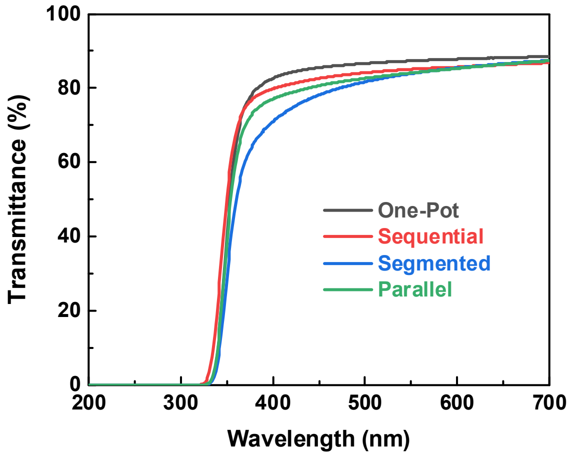

Figure S3. UV–Vis transmittance spectra of the F-PARs prepared by various feeding methods.

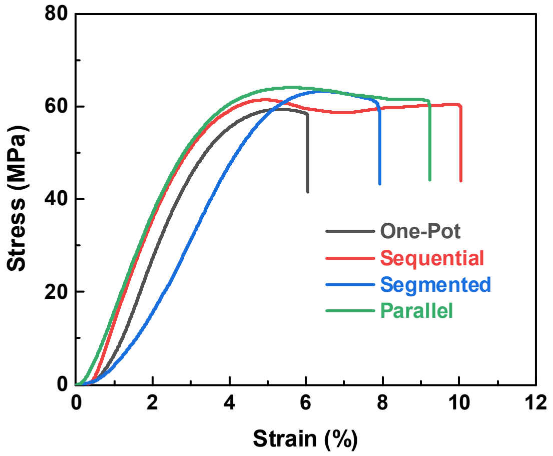

Figure S4. Stress–strain curves of the F-PARs prepared by various feeding methods.

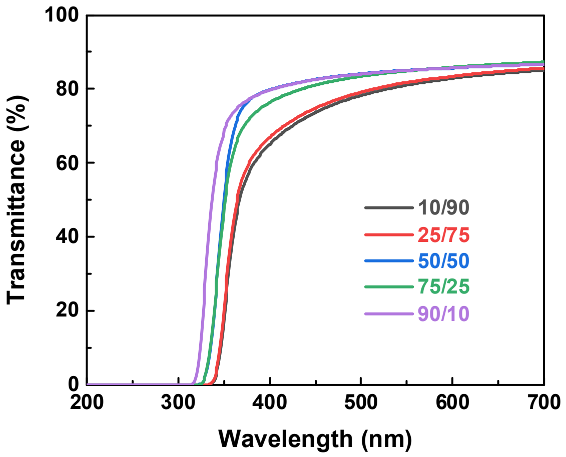

Figure S5. UV–Vis transmittance spectra of F-PARs with different BPAF/BPA molar ratios prepared by sequential methods.

https://doi.org/10.3390/polym18020267

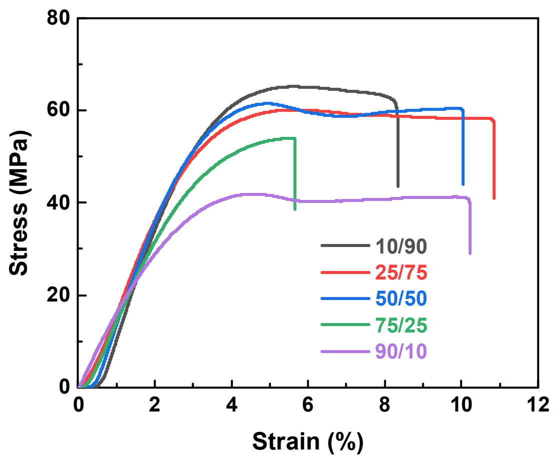

Figure S6. Stress–strain curves of F-PARs with different BPAF/BPA molar ratios prepared by sequential methods.
